# Visualising data science workflows to support third-party notebook comprehension: an empirical study

**DOI:** 10.1007/s10664-023-10289-9

**Published:** 2023-03-23

**Authors:** Dhivyabharathi Ramasamy, Cristina Sarasua, Alberto Bacchelli, Abraham Bernstein

**Affiliations:** Department of Informatics, University of Zurich, Zurich, Switzerland

**Keywords:** Notebook comprehension, Program comprehension, Data science code, Data science workflow, Workflow visualisation, Garden of forking paths, Software visualisation, Jupyter notebooks

## Abstract

Data science is an exploratory and iterative process that often leads to complex and unstructured code. This code is usually poorly documented and, consequently, hard to understand by a third party. In this paper, we first collect empirical evidence for the non-linearity of data science code from real-world Jupyter notebooks, confirming the need for new approaches that aid in data science code interaction and comprehension. Second, we propose a visualisation method that elucidates implicit workflow information in data science code and assists data scientists in navigating the so-called *garden of forking paths* in non-linear code. The visualisation also provides information such as the rationale and the identification of the data science pipeline step based on cell annotations. We conducted a user experiment with data scientists to evaluate the proposed method, assessing the influence of (i) different workflow visualisations and (ii) cell annotations on code comprehension. Our results show that visualising the exploration helps the users obtain an overview of the notebook, significantly improving code comprehension. Furthermore, our qualitative analysis provides more insights into the difficulties faced during data science code comprehension.

## Introduction

In recent years, data science has become a major resource in the decision-making process. This became undeniable during the recent COVID-19 pandemic, in which governments and scientists monitored and predicted the evolution of the spread of the virus using data science methods. To make their results reproducible, verifiable, and extensible by others (Randles et al. 2017), data scientists tend to share the code they create using computational notebook environments like Jupyter (2015).

However, several challenges exist in understanding and, consequently, reproducing the notebooks (Brandt et al. 2008; Pimentel et al. 2019; Merali 2010). Rule et al. (2018b) found that publicly available data science notebooks usually lack documentation. Undocumented third-party software code, in general, is challenging to comprehend (Francese et al. 2017; Letovsky and Soloway 1986; DeLine et al. 2005). Similarly, understanding a notebook without explanations from its author(s) can create confusion and misunderstanding (Rule et al. 2018b).

Furthermore, data scientists often present their results from the exploratory analysis, which are generally a result of choices determined by subjective reasons (Silberzahn et al. 2018; Schweinsberg et al. 2021), as an exploration of a single data analysis path. On the contrary, researchers have long advocated for the transparency of the *computational narrative*^1^ (Gelman and Loken 2013; Wacker 2017; Silberzahn et al. 2018; Rule et al. 2018a; Hill et al. 2016) i.e., making the data analysis process, decisions, and resulting artefacts including the dead-ends explicit, by documenting them. Gelman and Loken (2013) describes data analysis as a *garden of forking paths* and recommends documenting all of the alternative statistical analyses taken, successful or not, as each of them can highly influence the outcome (Schweinsberg et al. 2021).

In addition, while preliminary observations suggest that exploratory data science programming is iterative (Kery et al. 2019) and non-linear (Kery et al. 2017; Rule et al. 2018b), existing computational notebook environments like Jupyter allow data scientists to implement and record their analyses linearly. Because of this, data scientists often actively curate their notebook during its development by re-writing existing cell content, adding cell(s) in between other cells, or deleting existing cells in order to keep it concise and from growing. This behaviour also often leads to loss of information about their previous steps, exploration or (unsuccessful) alternative analyses.

Existing works on computational notebooks have proposed approaches that track the provenance and versioning history to keep a record of the analyses (Srinivasa et al. 2016; Pimentel et al. 2015, 2019). These approaches and their respective visualisations (Pimentel et al. 2015; Kery et al. 2019) to help reason about the analysis rely on the details (e.g., function definition (Pimentel et al. 2015)) of the source code and their execution as they occur linearly. However, keeping track of and foraging through a large number of source code-level changes is difficult for the data scientists (Srinivasa et al. 2016; Pimentel et al. 2015; Kery et al. 2019; Green and Petre 1996). Further, these approaches are limited to the analyses of source code and its related artefacts (e.g., data flow and control flow) and do not consider the non-linear nature of data science workflows and its steps. As a result, their visualisation does not represent the analysis in the form of *garden of forking paths*.

Prior work also shows that, due to active curation of the notebook, data scientists struggle to keep track of their mental model during development (Kery et al. 2017). However, there is a lack of research on how this affects the comprehension of data science code, particularly by third-party data scientists. The field of software engineering (SE) research extensively studied methods that facilitate program/code comprehension (Fjelstad and Hamlen 1979; Minelli et al. 2015; Corbi 1989; Ko et al. 2006; Storey et al. 1997a; Collberg et al. 2003), especially documenting for evolving software. Software visualisation tools, particularly software exploration tools “which combine graphical representations of software structures linked to textual representations of source code and documentation” (Storey et al. 1997a), are used to facilitate program understanding by forming a mental model of the software (Storey 1998). In order to facilitate the construction of a more accurate mental model during *notebook comprehension*, it is important to make the narrative as explicit and complete as possible, as indicated by Kery et al. (2018).

In this work, we first perform an empirical analysis on a set of data science notebooks in order to supplement the existing observations on the iterative and non-linear nature of data science programming that motivates this work. Further, we propose an explorable visualisation method that aims to enrich existing narrative information by elucidating the implicit workflow information in computational notebooks. Our interactive graph-based visualisation method represents and visualises a data science notebook’s (non-linear) workflow information in terms of forks and dead-ends. Additionally, we investigate how our data science workflow visualisation can help in *notebook comprehension* using a controlled user experiment.

The experiment is conducted with 35 data scientists who work as research scientists at a university or as freelancing data scientists in Upwork. During the experiment, each group of users was provided with different workflow information about (i) divergent paths, using the concept of forks and dead-ends described by Gelman and Loken (2013), and (ii) annotation of the data science steps present in the code. For the experiment, we implemented a prototype^2^ of the interactive visualisation plugin for Jupyter, named marg (which means “path” (Dictionary 2020)) - Narrative Exploration, that presents a static graphical representation of the underlying notebook’s workflow. We presented this graphical representation to data scientists together with the code as an aid to navigate and understand the code in the notebook. We further asked them to fill in a questionnaire where we asked questions that allowed us to measure comprehension accuracy, time taken to comprehend the notebook, and the usability of our solution.

The overarching goal of the work we present in this paper is to enable data scientists in their notebook comprehension activity through an explorable visualisation method.

Overall, the main contributions of this paper include:

- Empirical evidence, based on the analysis of 470 real-world Jupyter notebooks, shows that 81.1*%* of all the notebooks potentially follow a non-linear workflow in terms of the data science actions they implement, whereas 30.0*%* of them had more than three potential divergence points (Section 5.3). Further manual investigation of forks on 47 notebooks shows that, on average, at least about 0.9261 forks (i.e., a bit less than one) exist in a notebook.
- marg, a graph visualisation method to represent data analysis in computational notebooks and its implementation in order to visualise the notebook’s implicit workflow information (Section 6.2).
- Empirical evidence based on a controlled experiment with 35 data scientists that:
  1. Data scientists using marg reach a *statistically significant improvement* in the comprehension of a given notebook in terms of *comprehension accuracy* (Section 6.5.1).
  2. Data scientists in groups that had access to marg spent more time in answering the comprehension questions but *less time to get an overview of the notebook* given the maximum time scheduled for all groups was equal (Section 6.5.2).
  3. Based on the usability analysis of our solution, users receiving information about the data science step annotation gave a more positive score in terms of system usability than users receiving information about the workflow (Section 6.5.3).
  4. Data Scientists from all groups highlighted the lack of narrative text (including comments, analysis rationale, and interpretation of results) as a major obstacle in third-party notebook comprehension. (Section 6.6).
  5. We also discuss the strategies used and obstacles faced during a notebook comprehension task.

The remainder of this article follows the structure given here: Section 2 introduces the terminologies used in this article. Section 3 provides an overview of the related work, and the research questions are presented in Section 4. Section 5 describes the methodology, analysis, and results of the empirical study on data science code linearity. Section 6 details the methodology, analysis, hypotheses, and results of the user experiment studying the influence of explicit workflow information on data science code comprehension. Section 7 discusses and elaborates further on the insights we obtained from the user experiment and their implications. Section 8 concludes the article with final remarks and summarises the implications based on the insights from the user experiment for future research.

## Preliminaries: Terminology

In this section, we introduce the terminology used throughout this article. The terminologies are motivated by the literature and are restricted to the context of exploratory data science workflows in computational notebooks like Jupyter.

In an ideal scenario, a data science workflow (*SC*_*WF*_) is implemented through a series of executable data science activities^3^ from obtaining the data, exploring the data (in the case of exploratory tasks) and(-or) modelling the data (in the case of predictive tasks), and interpreting the results (Perkel 2019). In a computational notebook, this may include some or all of the following steps (Ramasamy et al. 2022): helper functions, load data, data preprocessing, data exploration, modelling, evaluation, prediction, result visualisation, save results, comment only. When connected sequentially, these steps comprise a data science pipeline.

A ***data science pipeline*** is a ***linear(-ised) workflow*** ($SC_{WF}^{linear}$) that follows a single path of sequential steps. Hence, a linear workflow $SC_{WF}^{linear}$ = < *s*_1_,…,*s*_*n*_ >, contains a sequence of steps *s*_*i*_, where *s*_*i*_ is a code fragment corresponding to a data science step. In Jupyter notebooks, these code fragments usually correspond to cells (Head et al. 2019; Jupyter 2015; Rule et al. 2018b). In an earlier study, we found that $\sim 76$% of the cells in DASWOW dataset contain a single data science step (Ramasamy et al. 2022).

In exploratory data science tasks, workflows are generally non-linear. A ***non-linear workflow*** (Kery et al. 2017; Patel et al. 2008) contains diverging paths that are referred to as *forks*. Every point at which a fork happens in a non-linear workflow is a *divergence point*. A non-linear workflow $SC_{WF}^{non-linear} = (S,E_{directed})$ is a directed acyclic graph of steps, where the steps *s*_*i*_ ∈ *S*, directed edge *e*_*j*_ ∈ *E*_*directed*_, and $e_{j} \subseteq S \times S$. A non-linear workflow composed of more than one fork, ideally, can be transformed into a set of pipelines by decomposing the workflow with respect to the divergence points.

A ***fork*** is a divergent path, starting at a divergence point in a data science workflow and provides a new, alternative, or complementary analytical path (Gelman and Loken 2013; Rule et al. 2018b). Hence, a fork is a step *s*_*i*_ that has at least two or more outgoing edges. While there can be multiple reasons for forking a data science workflow, some examples include modelling with a different feature selection, relaxing an assumption and recomputing a model, applying a different learning algorithm, and using another subset of the data.

A ***dead-end*** is a point in a data science workflow at which a set of steps taken in a data analysis does not (or cannot) advance (Patel et al. 2008). Hence, a dead-end step *s*_*i*_ will have no outgoing edges. Dead-ends are often the result of how data scientists repeatedly interact with the data, often in an explorative manner. For example, a dead-end would be the conclusion of a set of steps that lead to a hypothesis’ result, which turned out to be uninteresting or false. A dead-end can occur within a fork and may pave the way for another fork in the data analysis. While dead-ends are not necessary to reproduce the final result, they influence and guide the workflow of the analysis (Kandel et al. 2012) and may be crucial to understanding the rationale of the analysis.

***A computational narrative*** details a multi-modal account of a data science workflow that tells the story of ‘the data and the computations that process and visualise those data for a particular audience and context’ (Granger and Pérez 2021). It includes the source code (data science workflow) used for the task and its execution results (Granger and Pérez 2021) and narrative text about both the code and the results (Granger and Pérez 2021) including the rationale behind specific choices made throughout the task (Granger and Pérez 2021; Kery et al. 2017).

Hence, the computational narrative can be defined as a tuple *C*_*narrative*_ = (*SC*_*WF*_, *D*,*T*_*narrative*_,⋅), where *SC* is the source code, *D* is the data used including the input data *D*_*input*_, intermediate data *D*_*intermediate*_, and the output data *D*_*results*_, and *T*_*narrative*_ is the narrative text. Note that we have an inclusive view of data which includes structured datasets such as tables of graphs and unstructured data such as texts, images, as well as figures. Also, note that it may contain additional information (such as external supplementary files) not discussed in this paper (denoted with ⋅).

***A data science narrative*** consists of a linear or a non-linear ***data science workflow*** that implements the solution for a data-oriented task. Hence, a data science narrative *DS*_*narrative*_ = (*SC*_*WF*_,*D*,*T*_*narrative*_,⋅) is a type of computational narrative in which the source code *SC*_*WF*_ is composed of a set of data science steps.

***Computational notebooks*** allow implementation and documentation of computational narratives that can contain source code, execution results (outputs such as a plot, print statements etc.), natural language text, equations, media, hyperlinks, and any other visual representation, in a linear manner (Granger and Pérez 2021; Kery et al. 2017).

**Fig. 1:**
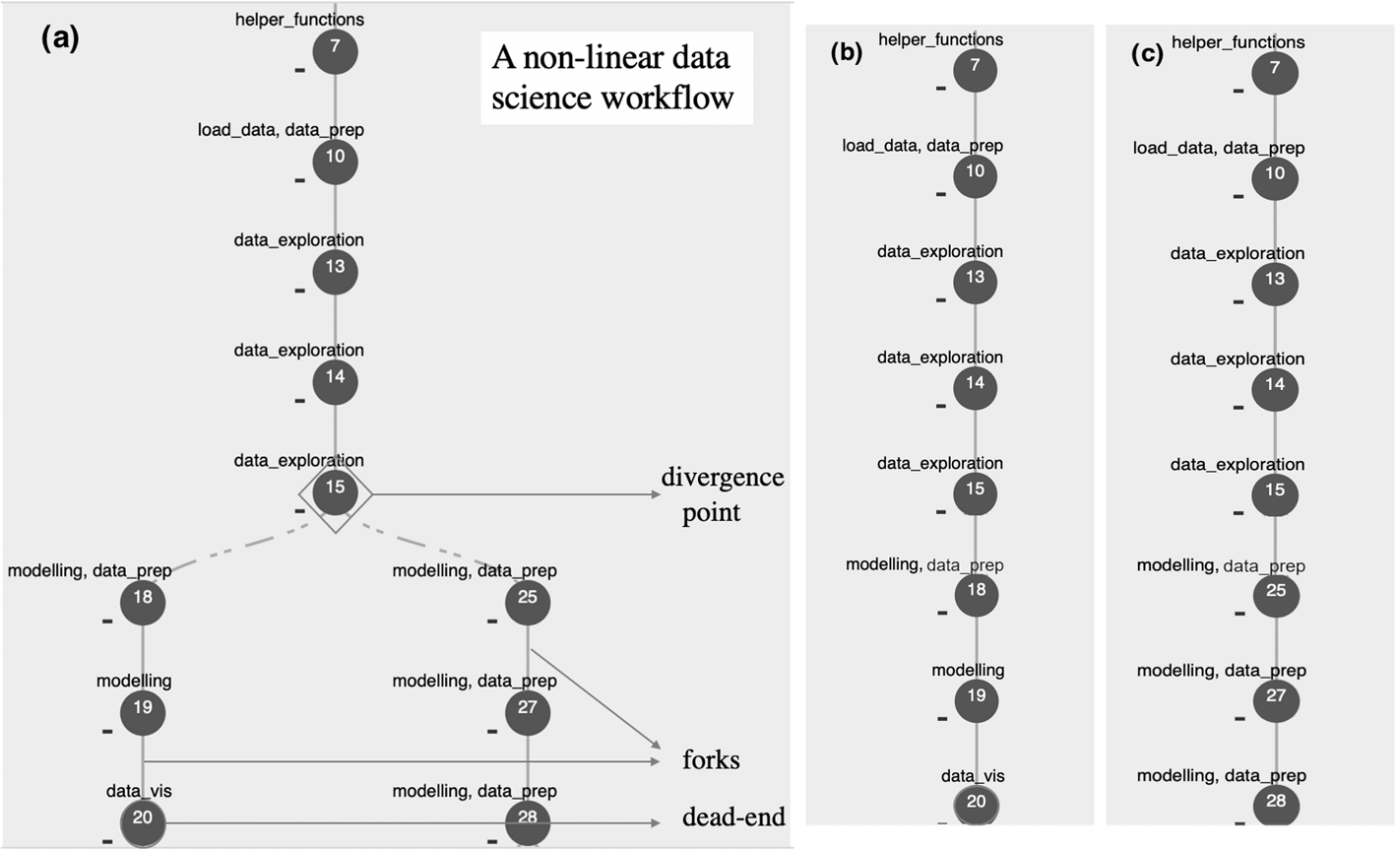
(1a) An example data science non-linear workflow of a computational notebook showcasing the divergence point, forks (using different modelling techniques) generating out of the divergence point, and a fork that may be leading to a dead-end. With respect to the computational notebook structure, each circle denotes a code cell identified with the number that denotes the order of the appearance of the cell and the data science steps it performs. Decomposed based on the divergence point at cell number 15, Figure (1b) shows a single pipeline of the non-linear workflow comprising the left fork and Figure (1c) shows a single pipeline of the non-linear workflow comprising the right fork

We illustrate the terminology using Fig. 1.

## Related Work

There is a large body of scientific work related to computational notebooks and, in general, data-intensive workflows. In this section, we summarise the most relevant works on computational notebook workflows, notebook comprehension activities, and software visualisation for comprehension.

### Computational Notebooks, Narratives, and Workflows

#### Existence of Non-Linear Workflow

A few studies have produced preliminary observations on the non-linearity of the data science workflow present in the computational notebooks (Rule et al. 2018b; Kery et al. 2018; Liu et al. 2020a; Pimentel et al. 2019). Rule et al. (2018b) and Pimentel et al. (2019) base their analyses on the execution numbers present in the notebooks to show that the notebooks have non-linear execution. However, more evidence is needed to generalise this finding because non-linear execution numbers can also occur due to various reasons, such as introducing a small change in the code. In the first part of our study, we expand on this and conduct an empirical study to investigate the existence of non-linear workflows in real-world Jupyter notebooks based on the data science step they perform.

#### Documenting the Narrative

Prior works on managing and documenting notebook narratives use the versioning history (Kery et al. 2017; Kery and Myers 2018; Head et al. 2019) and provenance (Pimentel et al. 2015; Koop and Patel 2017; Namaki et al. 2020; Macke et al. 2020) to track the analysis. However, keeping track of a large number of complex changes over an extended period is overwhelming for a human to interpret (Srinivasa et al. 2016; Pimentel et al. 2015; Kery et al. 2019; Green and Petre 1996). Another set of works uses the data flow information (Patterson et al. 2017), sometimes associated with a knowledge base of data analysis concepts (Patterson et al. 2017) to represent the analysis. The above-said works rely on the source code together with its execution providing a linear account of the analysis (based on a single trial or run). They do not address the challenge of representing a non-linear workflow of data science development. As Head et al. (2019) advocates for solutions that “add more sophisticated ‘layers’ to simplify and enrich notebooks’ narrative structure”, in marg, we focus on representing the high-level workflow information rather than the changes at the level of lines of code to enrich the narratives.

### Notebook Comprehension

Past research has explored how developers comprehend software programs and their relevant artefacts and built tools for supporting program comprehension. However, in the discipline of data science, the number of studies addressing this topic is still limited.

In exploratory programming tasks, Srinivasa et al. (2016) observed that programmers “build, refine, and use” a narrative, which is similar to a mental model-making process in traditional software engineering, to comprehend someone else’s code. Other studies also indicate that narratives are crucial for a third party to improve and extend an existing work (Kery et al. 2018). However, to the best of our knowledge, there is no existing research that studies how the workflow information within the narrative influences comprehension in the case of computational notebooks. In this work, we conduct an experimental study to understand how people, particularly third-party data scientists, comprehend someone’s data science notebook with the help of workflow visualisation. Additionally, we address the gap in data science comprehension studies by shedding light on the strategies and obstacles faced during the comprehension activity.

### Software Visualisation for Comprehension

Software visualisation methods have long been used to help understand software systems by visualising their structure, behaviour, and code. The most commonly used methods, particularly in small systems, are based on visualising graph representations (Ball and Eick 1996). Storey et al. (1997b) used graph visualisation techniques to explore a software’s structure and code. Similarly, Kienle and Müller (2010) produced an explorable high-level graphical view of software, whereas Systä et al. (2001) visualised static software artefacts as well as their dependencies as a directed graph.

In order to aid comprehension-related activities, different kinds of information related to the software are tracked and (or) extracted. DeLine et al. (2005) recorded activity and attention patterns that developers show while browsing software and used this navigation history to guide the third party through an unfamiliar code. Minelli and Lanza (2013) recorded and visualised the interaction of developers within the IDE to support comprehension, whereas Cornelissen et al. (2009), Pauw et al. (2001), and Systä et al. (2001) visualised program behaviour (execution) and structural attributes of the software (Cornelissen et al. 2009).

In the case of computational notebooks and, in general, scientific workflow systems, several works focus on visualising the provenance information (Pimentel et al. 2015; Kery et al. 2019; Patterson et al. 2017) of a data analysis code and their execution details (e.g., log data, data flow, control flow etc.) as they occur linearly. Alternatively, in this work, we focus on the workflow information and the implementation of data science steps in a notebook. As a result, we view the workflow in a notebook according to the data science steps and their divergences. This allows us to visualise the non-linear nature of data science workflows at a higher level of abstraction — at the level of decision and ideas.

In a different line of work addressing the multiple decision paths, Liu et al. (2020b) proposed a visualisation method called Analytic Decision Graph (ADG) to communicate decision processes for end-to-end analysis in the context of scientific publications and suggested that such a diagrammatic representation “linked to corresponding analysis code snippets (i.e., cells in a computational notebook)” will be helpful. In this paper, we propose a visualisation method^4^ similar to ADG that not only focuses on recording the decisions and rationale but also provides workflow information (with)in the context of data science activities. Our method is particularly adapted to capture and visualise the rich workflow of data science implementations in computational notebooks.

In our visualisation method, marg, we approach the workflow information for data science code at a higher level of abstraction — at the level of decision and ideas. Hence, we keep a record of the alternatives, unsuccessful approaches, and the workflow of the implementation itself. Furthermore, we also enrich the graph visualisation with other information, such as the rationale and the identification of the data science pipeline step through cell annotations. As a result, marg captures and visualises the data science notebook in the form of the *garden of forking paths* in order to elucidate the implicit workflow of a data science notebook. marg, with its explorable visualisation, also provides orientation cues (Thüring et al. 1995) (by identifying their current position in the workflow and focusing on the relevant code cell) and navigation facility to support the comprehension activity (Storey et al. 1997a).

Furthermore, we evaluate the visualisation for its ability to facilitate comprehension using an experimental study.

## Research Questions

As discussed in Section 3, while previous studies (Rule et al. 2018b; Kery et al. 2018) hint at the existence of non-linear notebook workflows, existing tools do not track the (work)flow of narrative at the level of ideas, methods, and decisions (Kery et al. 2019). Also, how the workflow information influences comprehension is not known. To address them, we structure our study around two research questions. The first research question focuses on empirically investigating the existence of non-linearity in computational notebooks based on the data science step they perform. 
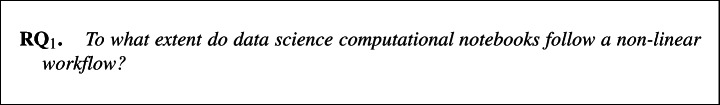


The second research question investigates to what extent elucidating the workflow information improves comprehension of third-party data science notebooks that contain analytical forking paths, as described by Gelman and Loken (2013). Note that while the initial work by Gelman and Loken (2013) and later by Schweinsberg et al. (2021) refers to a multi-user scenario in which the same hypothesis and data are given to several researchers who end up obtaining different solutions, we focus on augmenting data science implementation that contains forks, independently of the number of authors contributing to the code as even a single data scientist may (and should) explore multiple analytical paths (Gelman and Loken 2013; Steegen et al. 2016). 
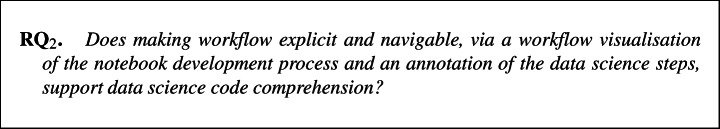


The following sections provide details on the research method used to answer these questions and discuss the results obtained. We make the dataset and analyses publicly available here: 10.5281/zenodo.7476596.

## Empirical Analysis of (Non-)Linearity in Computational Notebook Workflows (**RQ1**)

Our goal with this research question is to study the linear nature of the workflows present in data science notebooks w.r.t. data science steps they perform and augment the state-of-the-art findings with empirical evidence. To perform this study, we use DASWOW dataset^5^ that contains a set of 470 notebooks with each code cell annotated according to the data science step it performs.

### Data

We analyse the expert annotated dataset, DASWOW, from Ramasamy et al. (2022) containing a curated subset of notebooks that performed a data science task, randomly sampled from the $\sim 1M$ GitHub notebooks dataset by Rule et al. (2018b). DASWOW contains 470 notebooks with a total of 9,678 code cells and an average of 21 code cells per notebook. The annotation process was carried out by experts who manually inspected the notebook and annotated each code cell based on a 10-step characterisation of workflows in computational notebooks. As a result, each code cell in a notebook is assigned a primary label according to the primary step performed in the cell. Each code cell may also be assigned other labels called secondary labels if it contains other steps.

To get the workflows from the notebooks, we consider the sequence of data science steps based on the annotation information. Given the absence of order information for the secondary labels and that a majority ($\sim 76$%) of the code cells in the dataset contain a single data science step, we take into account the sequence of primary labels for the analysis.

### Methodology

In this section, we explain the strategy we used to empirically analyse the linearity of the workflows in data science notebooks based on the type of data science steps present.

We consider Jupyter cells as the logical separation of data science steps in a notebook. As a result, we view each notebook in the DASWOW as a sequence of data science steps (< *s*_1_,…,*s*_*n*_ >) occurring one after the other.

**Fig. 2:**
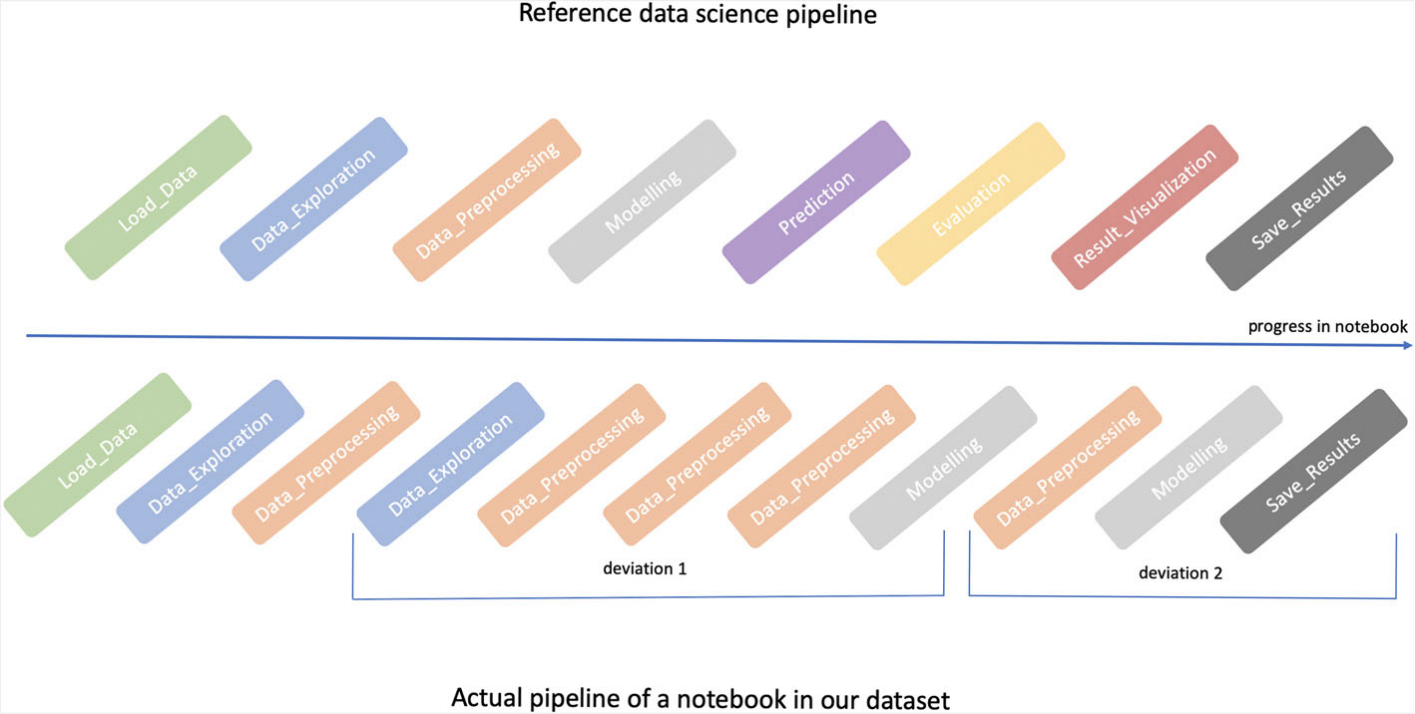
Sample workflow of a data science notebook characterised by the steps of the data science pipeline present in the cells of the notebook (lower half of the figure) compared to the reference data science pipeline (top half of figure). From left to right, we can observe the sequence of steps occurring in cells throughout a notebook. As seen in the figure, a notebook can have ‘potential’ forks/divergence points that (re)visit previous steps, making the sequence non-linear

For each notebook in the DASWOW, we then compare (i) the actual sequence of labels (< *a*_1_,…,*a*_*n*_ >) in the notebook to (ii) the sequence of labels of a reference data science pipeline $(<r_{step_{1}}, \ldots , r_{step_{8}}>)$. As a commonly prescribed data science pipeline, we specify the — sequential and linear — waterfall-like data science workflow as the reference pipeline that contains the complete set of data science steps for computational notebooks, from importing libraries to saving results (as identified by Ramasamy et al. (2022)). Figure 2 shows an example notebook workflow.

When comparing the actual list of steps in the subject notebooks, we ignore cells containing exclusively imported libraries or comments (i.e., cells annotated as helper functions and comment only). If consecutive cells contain the same data science step, we consider it as a single step as it indicates the code may be split among several cells.

We then analyse programmatically whether the actual data science actions in the notebook appear in the same order as in the reference waterfall-like data science pipeline, taking into account that some steps might be omitted (e.g., a data scientist might not have the need to clean the data in a notebook and therefore, there may not be a data preprocessing step in the notebook). Given that Jupyter notebooks follow a linear structure, we identify step $a_{i} (=r_{step_{i}})$ as a *divergence point* if the next step $a_{i+1} \in [r_{1},r_{step_{i}})$.

As a result, if the predefined order of progression is not followed, that is, when a previous step is (re)visited, then we declare the notebook as *potentially* non-linear and compute the number of divergence points (or ‘potential’ forks) occurring in the notebook. For example, if a notebook contains a data preprocessing cell after modelling, then that would account for one ‘potential’ fork. We do not, however, identify divergences that arise from non-consecutive cells. For example, in Fig. 2, we do not compare the first step of divergence 1 data exploration and the first step of divergence 2 data processing and account for another divergence point since users are likely to write code progressively in the immediate next cell following Jupyter’s linear structure. Also, as we consider consecutive cells containing the same data science step as a single step, we ignore divergences arising from them. For example, if two consecutive cells perform modelling but each of the cells applies a different modelling strategy, this will not be accounted. As a consequence, our results provide a lower-bound estimate for the presence of forks. We note that the identification of dead-end is not considered in this study.

We interpret the presence of divergence points as non-linearity and as a signal for potential forking paths resulting from the iterative and exploratory data science process. Using this method, we can control for the changes at a fine-grained level, i.e., re-executing a cell to fix programmatical errors. However, it is important to note that this method does not confirm the forks as this requires a better understanding of the developer’s intentions behind the code, which we do not have.

In order to tackle this and verify our findings, as a next step, we manually investigate whether the programmatically identified divergence points actually lead to forks using the information (code and markdown comments) in the notebook. To this end, we randomly sample a subset (10*%*) of notebooks. The first author of this paper then manually checked the programmatically identified divergence points to verify whether they led to forks. If we manually identify that a divergence point leads towards a different path of analysis (i.e., selecting different sets of different features to be modelled, applying different learning algorithms on the same set of features), we mark it as a ‘fork’. In the next section, we report the results from the programmatic analysis of divergence points, followed by the findings on forks from the manual study.

### Results

#### Programmatically Identified Divergence Points

The analysis shows that 81.1*%* of the 470 notebooks had at least one divergence point, and 30.0*%* of all the notebooks had more than three divergence points.

**Fig. 3:**
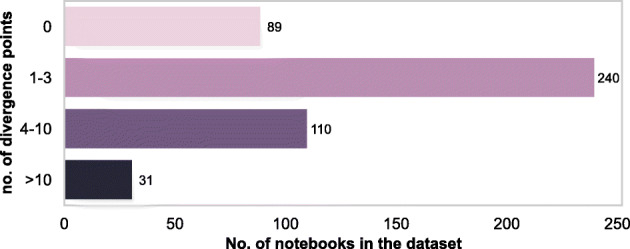
81.1*%* of the notebooks in the dataset had one or more divergence points

**Fig. 4:**
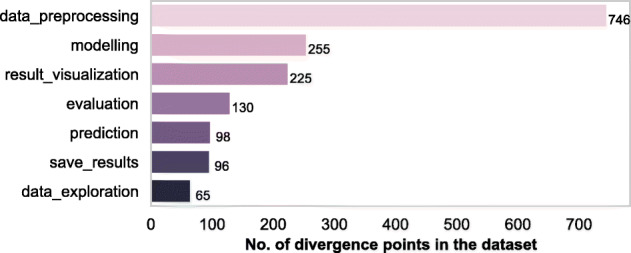
Where do potential forks occur? The majority of the divergences occur at the stages of data preprocessing or modelling during a data science task

Figure 3 shows the number of divergence points in a recommended linear data science pipeline in our dataset. We also found that the highest number of divergences occur at the data preprocessing step (see Fig. 4). These results provide more detailed insights into the non-linearity of data science code. In other words, apart from finding evidence to reinforce the growing perception that the data science development is explorative and iterative most of the time, we are able to untangle, with respect to the data science steps, some evidence for the existence of a certain amount of forking processes during the notebook development. The non-linear nature of the notebooks also shows that the data science development seems to follow the fail-fast agile methodology,^6^ which encourages the process of forking. That is, any direction of analysis (e.g., using a different statistical method or evaluation metric or data sample) taken by the analyst that does not lead to the desired result may be abandoned by proceeding to another, more promising direction leading to forks and dead-ends.

As Fig. 4 indicates, most of the divergence points happen during data preprocess ing. This is during the initial phase of the analysis when data scientists seem to be cleaning the data by trying various approaches.

#### Verification of *forks*

Through our manual investigation of a subset of notebooks, we studied 10.8*%* of divergence points in the DASWOW dataset. In total, 18*%*^7^ of the cells were programmatically identified as divergence points. Of these, we found that 24*%* of them are forks. Further, we found two false positive scenarios: one, where developers of the notebooks had generously used visualisations to explore and visualise the data and any (intermediate) results, which are identified as data exploration step. As a result, in many instances, data exploration as a label appears not only before data preprocessing but also after. Two, we found that the function definitions can appear in a different order compared to their original execution order; for example, a function defining the evaluation method may appear before the function defining the model. Taking into account the above two scenarios and eliminating the corresponding divergence points, we shortlisted 9*%* of the programmatically identified divergence points for further analysis. Of these, we found that 49*%* of them were indeed forks. This accounts for 4.41*%* of all the code cells being identified as a fork in the dataset. 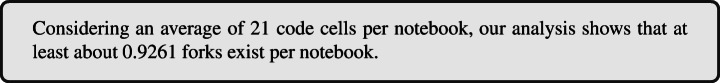


It is important to note that such divergences and their reasoning are hardly obvious in notebooks. As a result, an absence of the original developer’s mental model can lead to difficulties in the comprehensibility and reproducibility of the analysis, which motivates the second research question we address in this paper.

### Limitations and Threats to Validity

A potential validity concern is the representativeness and outdatedness of the dataset created by Rule et al. (2018b). In order to tackle this, we used DASWOW that contains a randomly sampled and manually curated subset of data science notebooks from Rule et al. (2018b). Further, as the dataset relies on publicly available notebooks on GitHub, we cannot rule out that the notebooks from other public platforms like Kaggle may differ as they are generally cleaned before sharing the solution (Quaranta et al. 2022) and are well-documented (Gil et al. 2010).

Another limitation is that the analysis considers only the primary labels given in DASWOW to extract the workflows of each notebook. A future study extending this investigation to include secondary labels may reveal further fine-grained insights.

Another possible concern could be that our study of non-linearity relies on DASWOW’s annotation. Also, given the snapshot view of the notebook, identified potential forks are not confirmed as, indeed, a fork. To address this, we manually verified the presence of forks in a smaller, randomly sampled set of notebooks. Further, given our manual analysis provide a lower bound to the presence of forks in an average-sized notebook, we suspect a higher frequency of forks in the case of larger notebooks. Our manual analysis also revealed further insights to improve the accuracy of programmatic identification of divergence points with additional rules (refer to Section 5.3).

## User Experiment on the Influence of Workflow Information on Data Science Code Comprehension (RQ2)

To investigate our second research question, we conducted a controlled user experiment where participants answered a set of comprehension questions. Using the experiment, we study the effectiveness of the visual representation of the workflow in notebooks (containing forking paths and data science step information) in helping data scientists navigate and understand data science code. In order to provide the visualisation, we first develop marg : *Narrative Exploration*, a plugin that visualises this workflow information and other workflow elements of a data science notebook.

Given the popularity of Jupyter python notebooks among data scientists (Perkel 2018), the selected environment for the notebook comprehension experiment is a Python notebook, executable within the Jupyter framework. The following subsections provide details of the data preparation tasks for the comprehension experiment, the implementation of the workflow visualisation plugin, and the concrete methodology followed in the user experiment. The relevant institutional ethics committee approved the experiments, and the participants explicitly consented to the usage and publication of anonymised results.

### Data Preparation: A notebook with a *‘garden of forking paths’*

For the notebook comprehension experiment, we use a data science task published in the literature, which “compared housing and rental prices in five major cities on the United States’ west coast” (Rule et al. 2018b). In order to generate a notebook with the solution to this task, exploring multiple analytical forking paths and generating information to capture its workflow information, we recruited two data science experts^8^ from Upwork, with experience in implementing data science solutions for clients and good reviews.

In order to capture all the relevant details whilst the data scientist implemented the notebook (that we would use as data in the target user experiment), we proceeded with a think-aloud approach, in which the data scientist explained the rationale of their decisions and provided information about the parts that we identify as forks and dead-ends. We informed the data scientists beforehand that the resulting notebooks would be shared with others and set the task’s duration to be 120 minutes. At the end of the task, we verified our recorded information, such as forks, dead-ends, and data science steps (as defined by Ramasamy et al. (2022)) with the developer in order to capture the information as accurately as possible.

While we hired two data scientists to have redundancy, the second expert’s dataset exploration was not satisfying and had only a minimal solution. As a result, we decided to use the notebook and information captured from one data scientist. The implemented notebook contained a few variable errors (e.g., using a variable that was later renamed in only some of the cells), missing inline output visualisations, and disorderly execution. However, we used the notebook provided by the data scientist as is, without any change, in the experiment to mimic real-world notebooks.

As mentioned in Section 2, the narrative of a notebook includes not only the explanatory information that is supposed to help to understand the story of the notebook but also the source code. We prepared the input data to represent the workflow information in the source code explicitly, along with the annotation of data science steps for the visualisation using the final notebook, information observed throughout the think-aloud task, other narrative information (additional description and rationale text), and the information gathered through the discussion with the developer. For the annotation of data science steps, we used the characterisation of data science workflows for computational notebooks provided by Ramasamy et al. (2022), which aggregates and extends state-of-the-art data science workflow characterisations. As a next step, we implement the visualisation for our plugin.

### Visualisation through marg : A narrative exploration plugin

The Jupyter plugin we devised and implemented visualises the data science code in a notebook and its underlying narrative elements, including *workflow information*, with the aim of improving comprehension. We designed the visualisation in an interactive way to allow the user to explore the narrative that emerged during the explorative development of a data science task by providing orientation cues and navigation facilities to aid the mental model-making process and support the comprehension activity.

**Fig. 5:**
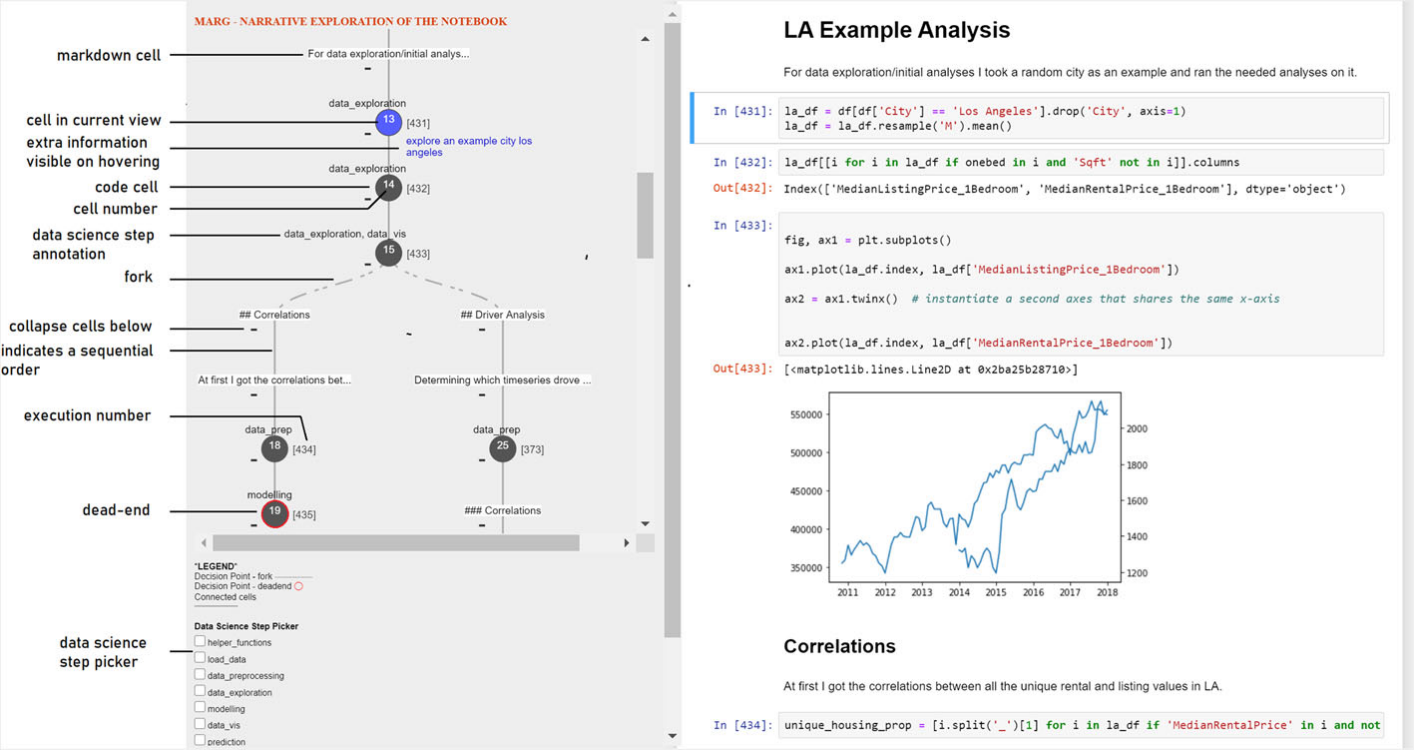
Components of the marg : Narrative Exploration plugin are identified in the figure. It shows the complete and original overview of the plugin with both tree workflow and annotation present. In the case of presenting only tree workflow, annotation is removed

Figure 5 gives an overview of the user interface provided via marg, augmenting the code of a Jupyter notebook. The plugin we implemented, once installed, is activated via the Jupyter toolbar. A short demo video of the plugin (marg-narrative-exploration- plugin.m4v) is available at https://doi.org/10.5281/zenodo.7476596. In its most complete version, the plugin presents the user with a graph visualisation on the left-hand side of the screen, which includes the workflow information and other elements, including data science step annotation.

Each cell in the notebook is visualised as a node (circular or rectangular, depending on whether they are code cells or markdown cells, respectively) and is identified by the cell number (or position w.r.t the sequence of cells in the notebook). It is accompanied by information about the order of execution it followed (presented in square brackets next to the node), the existence of forks, the data science step implemented in the cell (according to the categorisation we specified in Section 2), and additional textual information that explains the rationale followed by the developer of the notebook at each cell. A dashed line edge indicates a fork, and a solid line edge indicates a sequential set of cells. A red border around the node circle represents a dead-end. By double-clicking on a node, the user sees the workflow paths highlighted — either all of the paths (in the same colour) at the same time or individual forks (in different colour coding). When a cell node or a path(s) is highlighted, the corresponding cell(s) in the code is highlighted as well (in the same colour coding), providing a quick navigation access point. The user may also collapse/expand the nodes visualised in the workflow.

Additionally, users can select a list of data science steps in the checklist below to highlight the cells performing those steps in the graph. Together, the visualisation provides an overview of the data analysis process and serves as an enriched table of contents. The notebook itself is available on the right-hand side, which can be inspected and explored by navigating through the visualisation. Since our primary goal in this work is to empirically identify the kind of information that has the most positive influence on code comprehension, we focused on a static visualisation/representation for the experiment (i.e., the visualisation of the notebook’s original state is provided to the user and is not updated with any new changes in the notebook). The data (indicating the description of cells, their execution order, etc.) is provided as input to the plugin in JSON format.

**Fig. 6:**
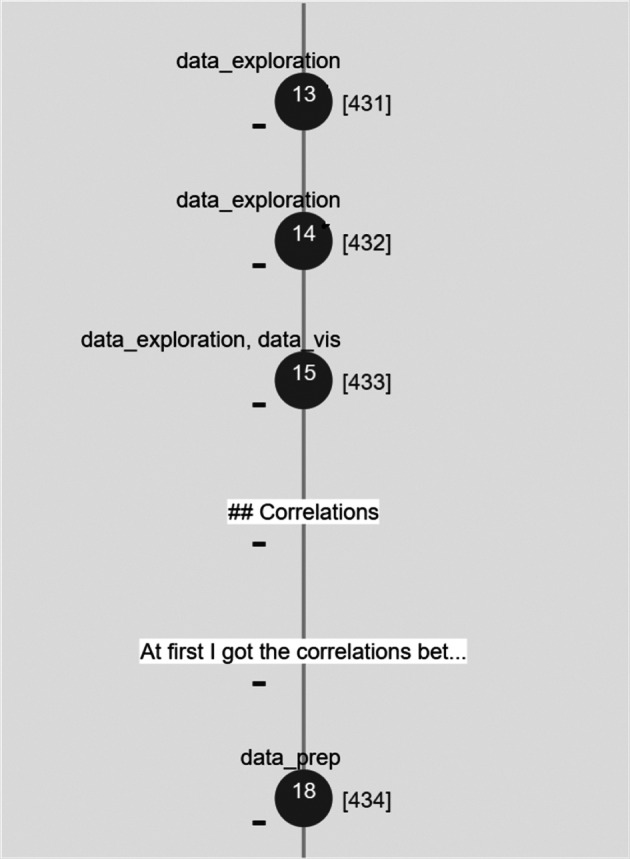
Linear workflow visualised with annotation. In the case of presenting only linear workflow, annotation is removed

**Fig. 7:**
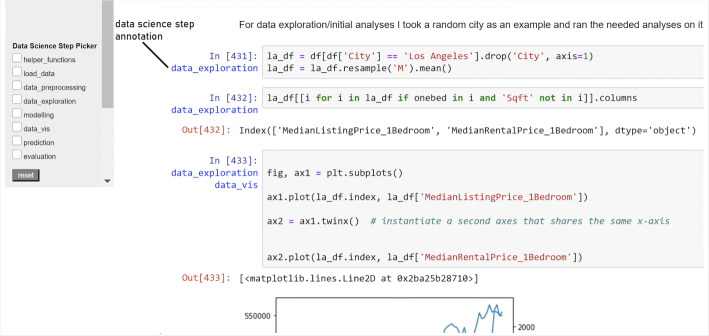
Visualisation presenting only annotation in the absence of workflow graph. Visualisation of only annotation is provided through the embedding of data science step information in the notebook

For the evaluation of marg, we created multiple versions of the visualisation information (see Figs. 6 and 7) based on two factors: the workflow information presented to the user is serialised either in a linear way (i.e., following the implementation order of cells and ignoring the existence of forks) or in a tree-based representation (i.e., making the beginning and the end of forks explicit based on the development process) and the data science step annotation, which is either available or not.

As a next step, we evaluate the effects of making the workflow explicit using a code comprehension task.

### Controlled Experiment Design

In this experiment, we investigate how marg can help in data science code comprehension by testing the participants against a set of questions. Specifically, we evaluate the workflow visualisation for two factors (independent variables):

workflow: workflow information as “garden of forking paths”: tree with forks and dead-ends (like shown in the upper left side of Fig. 5), linear progression (like in figure but only a linear representation of the cells shown in the order of the notebook), or no visualisation of the workflow.
annotation: information about the data science step each cell is performing is shown (as shown in Fig. 5 above the nodes; when no workflow is shown, then the step information would be added to the cell directly to the notebook).

#### Target Variables

We measured code comprehension in terms of the accuracy of the answers and response time, following the methodology proposed in Rajlich and Cowan (1997). *Accuracy* is the correctness of an answer given to a comprehension question when compared to the ground truth curated by the primary author of the paper and revised by a co-author. We calculate the comprehension score for each participant by adding the scores for eight comprehension questions (we explain more about the questions in a later section). We measure *Response time* as the time taken to answer each question. We record the time spent in two parts: prep time as the time taken to answer preparatory questions, and test time as the time taken to finish all eight comprehension questions.

#### Experiment Groups and Environment

We designed the experiment in such a way that the components of the visualisation ({workflow} and {annotation}) can be studied to discern the individual effects and their practical usefulness. The result is a three-by-two study (workflow: tree/linear/none; annotation: step-type/none) leading to a control group and five treatment groups (see Table 1).

**Table 1:** Experimental Design: 3x2 factorial setup of workflow and annotation modes leading to 6 experimental groups

		workflow: Workflow Visualisation		
		none	linear	tree
annotation:	none	control (5)	linear (5)	tree (10)
Data Science Step	annotated	DS (5)	linear.DS (5)	tree.DS (5)

Each group consisted of five participants to begin with. We added an additional five participants for the “tree” group in order to address the large dispersion of results (a higher standard deviation compared to the other groups) we found from the initial set of participants. As a result, the experiment had 35 participants in total and produced a setup where a total of 10 participants received “linear” workflow information, 15 participants received workflow in terms of forks and dead-ends (“tree”), and 15 participants received data science step annotation. The information shown to each group is given below:

- Control: notebook
- Linear: notebook, a linear workflow visualisation
- Linear.DS: notebook, a linear workflow visualisation accompanied with data science step annotation
- Tree: notebook, a tree-based workflow visualisation in terms of forks and dead-ends
- Tree.DS: notebook, a tree-based workflow visualisation in terms of forks and dead-ends accompanied with data science step annotation
- DS: data science step annotation

We set up the experiment environment in our local server with Jupyter and the MARG plugin with the corresponding information mode already installed. We allowed the participants to access the environment, including the experiment files (notebook and the dataset used in the notebook), remotely for the experiment duration. We also set up the survey questions and shared them with the participants during the experiment.

#### Participants

We recruited participants using various channels such as Upwork (16) and University contacts (19) and randomly assigned them to one of the groups shown in Table 1. Their highest attained educational degree varied between a high school diploma (5.7%), a bachelor’s (40%), a master’s (51.4%), and a doctorate (2.9%). All of them were either data scientists or data science students and had practical experience in python programming and Jupyter and had worked on data science tasks before. We provide further details on the participants’ experience in the Appendix (refer to Appendix Appendix).

#### Comprehension Questions

At the beginning of the experiment, participants received a set of general questions about the data science processes. We then followed the methodology suggested by Siegmund (2016) to devise the main comprehension task. We considered a set of 8 comprehension questions to assess the participant’s understanding of the data science notebook developed by a third-party data scientist. We devised these questions to test three dimensions of comprehension: navigation, understanding, and modification. We provide the list of questions and their format in the Appendix (refer to Appendix Appendix). We displayed them in the same order to all the participants across groups. Additionally, we also asked a preparatory question (Question 1) in the beginning in order to allow the participant to familiarise themselves with an overview of the notebook. Questions 2-5 tested the understanding of the notebook by asking questions like the number of features and the modelling technique used. Question 6 dealt with navigation by asking about the orientation of a cell, and Questions 7-9 tested the ability to make modifications to the notebook. We finished the task with qualitative questions on the comprehension process. Given its subjective nature, we always awarded a full point to Question 1 unless there was no attempt to solve it. We evaluated the answers to the other questions against our answer key. We awarded a score of 1 to each accurate answer and 0 to an incorrect answer. If a question is open-ended, we checked the answer (if available) manually in order to ascertain that the answer is their own and already not available in the presented notebook (e.g., a modelling technique that is not already showcased in the presented notebook). At the end of the experiment, we asked the participants to rate their comprehension of the notebook and answer a set of questions about their experience (comprehension strategy, issues and difficulties faced). Participants who received the marg plugin support also answered a usability test (Brooke 1996) (System Usability Scale (SUS)) on the plugin and its features. We report the complete list of questions (including the SUS questions used for relevant groups), instructions, the description of the task (refer to Appendix Appendix), and the reference answers for comprehension questions (refer to Appendix Appendix) in the Appendix.^9^

#### Experiment Setup

To answer the questions, participants were able to interact with the notebook and the additional visualisations (except for the control group) about the code according to their experimental group. Each participant received a reward of 25$ for participation and a 1.5$ bonus per accurate answer. Every participant spent about 2 hours on the experiment, including a time limit of 90 minutes (arrived at using a pilot study) for answering the comprehension questions, including the preparatory question. The participants were allowed to use resources freely as they would usually do in a regular comprehension task.

#### Experimental Procedure

We scheduled the experiment individually for each participant using a video call. We recorded the screen-share/audio of the experiment for any re-verification later. We destroyed the videos after scoring the questions in line with ethical guidelines. First, they answered generic questions about their knowledge of data science processes. The participants then watched the video of the notebook environment setup (including the explanation of the plugin features whenever applicable) and were then provided with 10 minutes to try the setup with an example notebook. After the video, the participants received the notebook with the plugin showing the features according to their treatment group — except for the control group, which only got the notebook — and were asked to answer the preparatory question and comprehension questions. The participants were allowed to search or use any online resources such as Google, StackOverflow Etc. during the task. Finally, the participants answered questions about their comprehension strategy, experience, and usability of the plugin (if applicable).

#### Hypotheses

Our main hypotheses focus on the improvement in comprehension accuracy and time: 
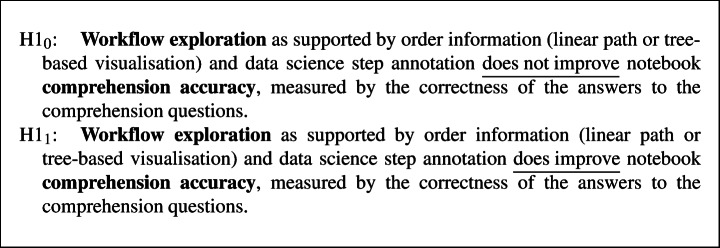

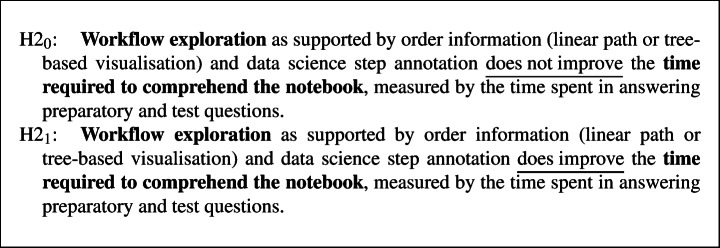


### Methodology

For the inferential analysis, we employ parametric two-way ANCOVA to study the two factors: workflow and annotation and their interaction effects. While conducting the analysis of variance, we control for the effects of prior experience with data science, Jupyter notebook, python programming, and programming in general (at the subject level) and the plugin features (at the environment level) by including these terms in the model. For the qualitative analysis, we employ the in-vivo qualitative coding strategy.

### Results

Our user experiment, which assesses the influence of different workflow information on data science code comprehension, led to both quantitative and qualitative results. We begin our quantitative analysis by performing ANCOVA tests on the comprehension score achieved through answering the comprehension questions. Then, we examine the response time taken to answer the questions and the usability study results. We continue by discussing the qualitative results regarding the comprehension experience, usability factors, and practices relevant to linearity.

#### Data Science Code Comprehension - Accuracy Score

Table 2 shows the summary statistics for the comprehension score over different groups. Tree.DS has the highest mean comprehension score among all the groups.

**Table 2:** Summary statistics for the comprehension score

group_name	N	Mean	SD	SE	95% Conf.	Interval
DS	5	3.0	1.225	0.548	1.800	4.200
Control	5	3.2	0.837	0.374	2.380	4.020
Linear.DS	5	3.2	1.483	0.663	1.746	4.654
Tree	10	3.6	1.430	0.452	2.666	4.534
Linear	5	4.2	0.837	0.374	3.380	5.020
Tree.DS	5	5.4	1.140	0.510	4.282	6.517

**Fig. 8:**
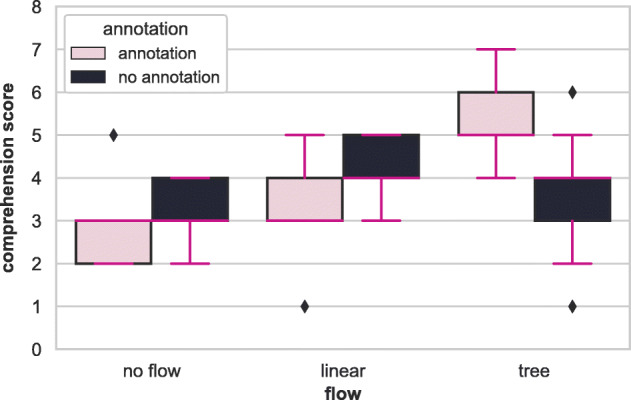
Box plot showing comprehension score results over workflow & annotation factors

**Fig. 9:**
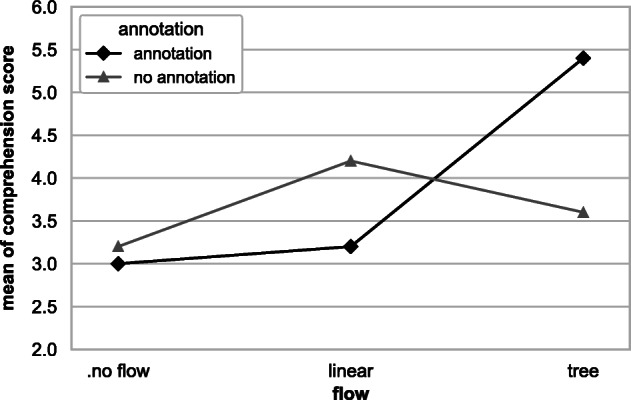
Interaction plot for workflow & annotation factors. Workflow visualisation produces an improvement in comprehension score (particularly when it is a tree workflow representation), and it is further improved by the presence of annotation


Figure 8 shows a box-plot of comprehension score results over {workflow}& {annotation} factors. We report the statistical significance and the cohen’s f (Cohen 1988) effect size from our ANCOVA models using partial *η*^2^, which corresponds to the proportion of variance accounted for by each main effect, controlling for all other effects. A two-way ANCOVA test for the {workflow} and {annotation} factors shows a statistically significant difference with respect to interaction effect (*F* = 5.6,*p* = 0.01) with an effect size of 0.7 (large (Cohen 1988)). The factors {workflow} and {annotation} individually achieve *F* = 1.83,*p* = 0.18 with an effect size of 0.4 and *F* = 0.68,*p* = 0.42 with an effect size of 0.2 respectively. An interaction plot for the comprehension score is shown in Fig. 9. Based on these results, we conclude that:

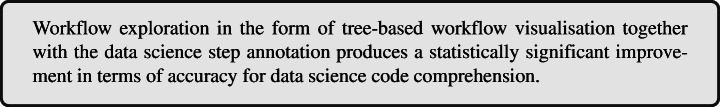


Given the statistically significant interaction, we utilise the simple effects of the t and {annotation} factors. This corresponds to the mean comprehension scores presented in Table 2. The increasing order of the performances of the groups on comprehension is *DS, Control, Linear.DS, Tree, Linear, Tree.DS*. Groups that received linear or tree {workflow} performed equally or better in comprehension scores than the Control group. The Linear group performs well without annotation, and the Tree group performs better with the annotation information. Tree.DS group achieved an improvement of $\sim 69$% over the Control group on mean comprehension score. However, groups that received either none or linear {workflow}, which is Jupyter’s original workflow information, did not gain from step {annotation}. We suspect the {annotation} information is more helpful when used with the navigation capability of the plugin since users can navigate to the desired cell corresponding to {annotation} without excessive scrolling.

#### Data Science Code Comprehension - Response Time

Table 3 shows the summary statistics for the total time taken to finish comprehension questions over different groups.

**Table 3:** Summary statistics for the comprehension time (in sec)

group_name	N	Mean	SD	SE	95% Conf.	Interval
Control	5	1594.498	441.1642	197.2946	1046.7203	2142.2757
DS	5	2111.456	1235.3200	552.4519	577.6036	3645.3084
Linear.DS	5	2203.660	674.0463	301.4427	1366.7210	3040.5990
Tree.DS	5	2246.460	476.8663	213.2611	1654.3523	2838.5677
Tree	10	2249.021	437.4145	138.3226	1936.1135	2561.9285
Linear	5	2471.088	1008.9968	451.2371	1218.2530	3723.9230

**Fig. 10:**
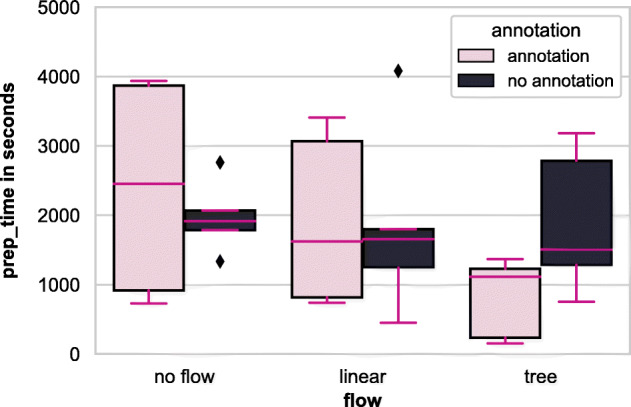
Prep time among workflow & annotation factors

**Fig. 11:**
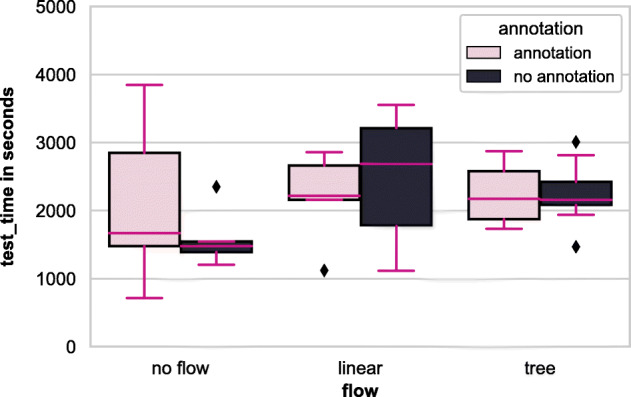
Test time among workflow & annotation factors

Figures 10 and 11 show an overview of the prep and test completion times, respectively, of all the groups. During the experiment, we found that 77% of the participants spent one-fourth of their time, and around 37% of the participants spent half of their total time in the preparatory question getting an (initial) idea of the notebook.

A two-way ANCOVA test on prep-time and test-time produced results for {workflow}, {annotation}, and interaction that are not statistically significant, thus meaning that we cannot reject the null hypothesis. In other words, we could measure no significant difference in the time taken to understand the notebook and answer the comprehension questions among the experiment groups. We also analysed the time taken for the test questions in conjunction with the answers’ correctness but did not find any statistically significant results. However, from Figs. 10 and 11, we can see a general trend for the preparatory question that having {workflow}, particularly tree, and {annotation} helps in acquiring a general overview of the notebook quickly. As a result, we conclude that: 
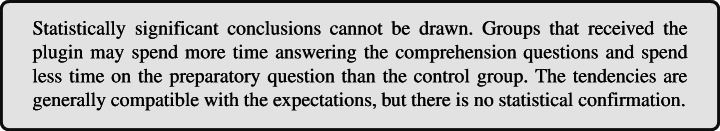


#### marg : Usability

The System Usability Scale (SUS) analysis on the plugin showed that the participants generally reported better usability when {annotation} is present. The plugin had an overall SUS score of 76.5*%*, which is a good score for acceptability (Bangor et al. 2008). Table 4 shows the SUS score over the components of the workflow exploration plugin using a table of means analysis. The scores are indicative, given the differences were not statistically significant (for *p* = 0.05).

**Table 4:** Usability score for marg : Narrative exploration plugin

	Annotation.absent	Annotation.present	**Marginal mean**
No flow	−	79.500	**79.500**
Linear	71.000	80.000	**75.500**
Tree	76.750	78.000	**77.375**
**Marginal mean**	**73.875**	**79.167**	**76.521**

The annotation factor, in general, has a better score than the workflow factor. The primary concern for workflow factor is that it takes nearly half the size of the available window view, thus hindering the normal Jupyter notebook experience

Considering the comprehension score achieved, having both tree-based {workflow} and {annotation} has a good usability score (78*%*). We hypothesise that having only {workflow} information could have led to a cognitive load even though it produced a better understanding of the notebook’s original purpose and intent (as is reflected in the comprehension score). In the case of tree {workflow}, it requires the participant to think in terms of the *garden of forking paths* that marks a deviation from their original Jupyter notebook flow. Using open questions, participants expressed that they did not feel linear {workflow} added additional benefits since the workflow presented is redundant to the linear {workflow} already provided by the Jupyter notebook (also reflected in the mean usability score of 75.5*%*). The presence of {annotation} had a higher usability score (79.2*%*) compared to the absence of it (73.9*%*). We would like to note here that the participants who had {annotation} information also reported a better comprehension of the notebook (refer to Section 6.6.1), showing its usefulness. We report the qualitative results on the usability of the plugin in Section 6.6.2.

### Qualitative results

The goal of our qualitative analysis is threefold: first, we analyse the participants’ code comprehension experience, namely their perceived comprehension score, the strategies they followed to understand third-party data science code, and the difficulties they encountered in this process. We further analyse the obstacles to comprehension that the data scientists in our experiments reported. Second, we identify the factors that contributed positively and negatively to the usability of our proposed solution. Third, we report the participants’ results on their own data science workflow development practices in the context of the ‘garden of forking paths’.

#### Comprehension Experience

##### Perceived Comprehension

**Fig. 12:**
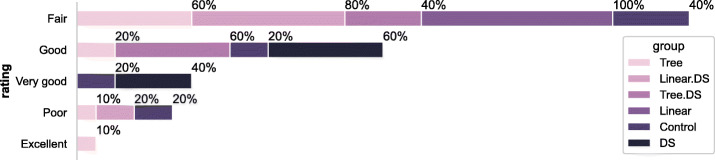
Perceived comprehension rating

We asked the participants to rate their comprehension using a 5-point Likert scale ranging from *1-Poor Comprehension* to *5-Excellent Comprehension*. Figure 12 shows a summary of the participants’ ratings as they are perceived by experiment groups. 11.4% of all participants rated their comprehension of the notebook as *good* or *excellent* while more than half of the participants (54.2%) stated that their comprehension was *fair*. We found that perceived comprehension is highest in the group that received only the annotation, whereas it is lower in the groups that received a linear {workflow}. Participants who received a tree {workflow} stated a higher perceived comprehension, with or without annotation.

Our analysis shows that a user’s perception of how well they comprehend the notebook may not necessarily reflect in the performance. This is particularly reflected by the group that received only the annotation. While their corresponding comprehension score was the lowest (refer to Section 6.5.1), they (53.3*%* of the participants who received {annotation}), however, had generally reported a higher comprehension (‘Good’ or ‘Very good’) of the notebook. Based on our analysis of further qualitative comments, we suspect that the mismatch is because users seemed to prefer information that may be less cognitively heavy despite their value of being useful.

##### Strategies for Data Science Code Comprehension

We asked the participants about the strategy they followed to comprehend the notebook, giving them two fixed alternatives as identified by Littman et al. (1987) (which differentiate between skimming and carefully reading all the code) and an open-ended third option. 68.6% of the participants indicated that they followed the strategy of *“skimming through the code and then, navigated through the code snippets as needed”*, and 25.7% stated that they followed the strategy to *“first read the code completely to have an overview”*. This shows that the majority of the data scientists, when they read a third-party data science notebook, are primarily interested in getting an overview of it. Finally, 5.7% said they would employ a different strategy, which corresponded to using both the strategies mentioned above.

##### Obstacles to Data Science Comprehension

We asked participants in an open-ended question to elaborate on the difficulties they encountered in the process of understanding the data science code in the notebook. We implemented an in-vivo coding methodology (Saldaña 2015) to process the participants’ answers and to extract and categorise the significant obstacles to data science code comprehension. Below, we thematically elaborate on the difficulties sorted by their frequency of occurrence:

1. *narrative text*: A lack of enough narrative text (e.g., descriptive information in the form of comments, rationale, and result interpretation) leads to difficulties in comprehension. A total of 65.7*%* participants reported this difficulty.
2. *code*: Code-related issues faced during comprehension include the presence of bugs, poor naming of variables, unclear data flow through the variables, and missing outputs. A total of 54.3*%* participants reported this difficulty.
3. *data*: Data-related issues include a lack of relevant analysis for the task, including a lack of sufficient exploration, analysis, and visualisation of the input data and its features. A total of 45.7*%* participants reported this difficulty.
4. *coding style*: Style-related issues during data science code comprehension include abnormal density and redundancy of the code. It also includes different programming styles both in terms of structuring the analysis and in general. A total of 42.3*%* participants reported this difficulty.
5. *knowledge of methods and algorithms*: This includes a lack of knowledge of certain functions or methods used as a result of the participant’s experience and expertise. A total of 25.7*%* participants reported this difficulty.
6. *structural*: At the notebook level, this includes excessive scrolling needs, non-linearity and logical disconnectedness in the code. At the cognitive level, a lack of mental model-related information contributes to structural issues. A total of 25.7*%* participants reported this difficulty.
7. *time*: The time given for the comprehension activity was considered insufficient by a few participants. A total of 17.1*%* participants reported this difficulty.

**Fig. 13:**
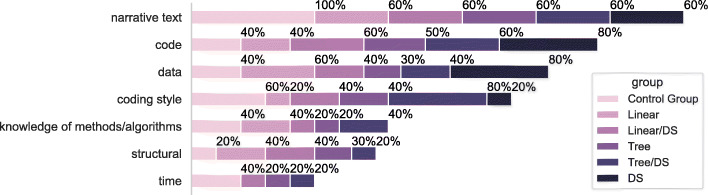
Obstacles in data science code comprehension as reported by the % of the participants in each of the groups. The majority of the participants, particularly from the control group, commented on the lack of narrative text. The other top difficulties were errors in the source code itself and a lack of detailed analysis of the data. The least of the concerns was the time constraint

Figure 13 shows the frequency of these obstacles in each of the experimental groups. Participants from the control group indicated that lack of enough narrative text and the need for more time were the major obstacles in the comprehension task. Coding style was the primary concern for groups that received {annotation} and tree {workflow}. Groups that received only {annotation} primarily reported obstacles related to data and code. Groups that received linear {workflow} reported obstacles related to structure. For groups that received {annotation} and tree {workflow}, coding style was the primary concern.

#### Usability Factors for our Plugin

**Fig. 14:**
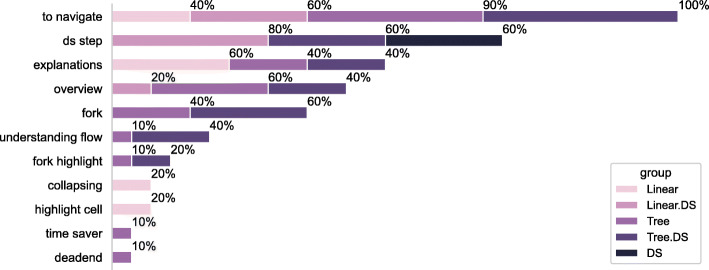
Participants’ remarks on what was the plugin helpful for?

We present here a qualitative analysis of the participants’ experience with the plugin and its usability. From Fig. 14, the top features that participants found as most **helpful** are: being able to navigate to a particular cell (63.3*%*), distinguishing cells based on their step in the data science pipeline (33.3*%*), the visualisation of the forks (30*%*) providing an overview, and the presence of additional explanations or rationale provided for a particular cell (30*%*). The participants mentioned navigation was beneficial in the presence of a tree {workflow}, possibly because it condenses the depth of the notebook. Groups (60*%* of the participants in the Tree group and 40*%* of the participants in the Tree.DS group) that received a tree {workflow} also indicated that the plugin helped them to get an overview of the notebook and understand the flow of analysis. 10*%* of the participants that belonged to the Tree group did not feel they had a use case for highlighting the paths in the tree. Participants (30*%*) indicated the ability to collapse certain parts of the tree as the **least useful** feature as they rarely had the need. We suspect this might change if the notebook becomes more complex, with many paths and dead-ends.

The **difficulties** (reported by 26.6*%* of the participants) in using the plugin mainly stemmed from the large size of the window it occupied. In addition, one participant mentioned that double-clicking a cell to visualise its path was difficult using a touchpad. Participants also expressed their **desire** to have more information (e.g., thumbnail of the actual cell content, output from the last execution of the cell) for each node in addition to the cell number (6.6*%*) and expected some support for handling variable flow (6.6*%*).

#### Common Data Science Practices Relevant to Linearity

Participants also answered additional questions related to data science development that we posed to complement our findings of data science code linearity (Section 5.3). We asked them whether they explore alternative paths (forks and dead-ends) when implementing data science code. Most participants ($\sim 85$%) stated that they usually try different, alternative solutions when approaching a data science task. This insight corroborates and triangulates our findings on the potential existence of forks and dead-ends in the notebooks. A majority of the participants ($\sim 60$%) also mentioned that the data science development follows either agile or iterative methodology, which is similar to our empirical finding from Section 5.3.

### Limitations and Threats to Validity

In this section, we discuss the limitations and potential validity concerns of our comprehension experiment, including the data preparation activity.

Validity concerns arise primarily from generalising the results of a single notebook concerning one field to a broader context. However, we used an existing data science problem from the literature in order to mitigate this issue. Also, a single data scientist developed the notebook; hence, that data scientist’s knowledge, expertise, and coding style are potential confounding factors. Therefore we cannot rule out that findings with a different notebook implemented by a different data scientist may diverge.

A second limitation concerns the participants’ lack of experience and knowledge about the plugin and its features. To mitigate this aspect, we first introduced the notebook environment and plugin (wherever applicable) in a short video and then we allowed the participants to familiarise themselves with the setup using an example notebook. We tried to minimise learning effects by introducing a preparatory question that allowed the participants to get familiarised with the notebook before answering the comprehension questions. Nonetheless, it is unclear how longer-term effects would factor into our results.

A further confounding factor of our experiment that could have affected our results is the participants’ perception and expectations of coding style and what is readable. Controlling for coding style expectations would require both a larger set of subjects and notebooks (with varying coding styles) — an investigation we intend to undertake in the future.

We allowed the participants to read and execute the notebook as they would typically do in a comprehension process. On the one hand, this experimental choice mimicked the real-world comprehension activity closer. However, it could have led to participants changing the original notebook before the comprehension questions were accurately answered. To avoid this, we placed the modification questions at the end such that the participant did not significantly change the original notebook.

Moreover, as stated above, in this experiment, the plugin provides users with a static representation of the notebook’s workflow. Hence, if users were to implement any change in the code cells that affects the workflow, their change would not be updated in the workflow graphical representation. Our rationale for this decision was to investigate the usefulness of this approach first and implement the dynamic extension of the plugin subsequently, taking into account the findings of this experiment.

Finally, we identify a generalisability limitation. The number of participants in each of the groups of our controlled experiment is limited. After analysing the initial set of results, we decided to limit the standard deviation below 2 in the comprehension scores of the groups. For that purpose, we added an additional set of participants in the case of the Tree group, which had a higher standard deviation in the initial set of results.

## Discussion

In this section, we further discuss the insights gained from the data science comprehension experiment and potential challenges and opportunities for future research.

### Comprehension Strategies in Data Science

While systematic studying of the program (a traditional piece of software) has been linked to success in activities involving modification of the program (Littman et al. 1987), a majority of the participants in our experiment preferred to skim through the code and then navigate through the code snippets as needed (refer to Section 6.6.1). Littman et al. (1987) showed that this strategy allows users to focus on a localised part of the code when navigation becomes critical to reaching the block of code quickly. This is expected as Jupyter notebooks have navigation difficulties due to the complex and linear structuring of the code (Kery et al. 2018, 2019). But at the same time, when the user applies skimming as a strategy to study the program, it results in weak mental models during comprehension and, as a result, does not lead to success in maintenance tasks (Littman et al. 1987). This has implications for the development of support tools for comprehension and maintenance of data science code.

Using our visualisation tool, we alleviate some of these difficulties. One, the tool allows the users to find and navigate directly to code blocks that perform a particular data science step without excessive scrolling and searching. Two, the tool supports the creation of a mental model by providing an overview of the notebook. However, there is still further potential for tools that support the comprehension activities in computational notebooks, for example, to allow the user to study the program in a systematic and detailed manner in conjunction with the workflow structure.

### Supporting the Development of Exploratory Computational Notebooks

We also found that users had varied opinions on what is a well-written code, similar to the findings in traditional software fields (Hulkko and Abrahamsson 2005; Begel and Nagappan 2008). A coding style that does not conform to their expectations created difficulties in the comprehension of the notebook. Examples include writing and structuring code blocks differently or declaring variables that do not follow the standard naming conventions. However, not all data scientists are always familiar with standard coding conventions (Kim et al. 2017; Wang et al. 2019).

By augmenting previous guidelines for making scientific analyses reproducible (Wilson et al. 2014), Rule et al. (2019) developed a set of ten rules in 2019, particularly for computational notebooks like Jupyter. It advocates simple suggestions like modular coding and accomplishing a single task per cell to improve computational notebooks. Similarly, Pimentel et al. (2019) developed another set of best practices in another study and consequently developed a Jupyter extension called Julynter (Pimentel et al. 2021) that provides suggestions based on these practices to improve a notebook’s reproducibility. In another study, Quaranta et al. (2022) adapted the existing practices, particularly for the collaborative development of notebooks. Quaranta et al. (2022) also found through an interview study that experienced data scientists followed a majority of these best practices in a collaborative context. However, they also found that these practices are difficult to follow in the case of exploratory data analysis, where notebooks contain non-linear exploration. As a result, data scientists, including experts, face difficulties in balancing the effort to create quality notebooks while exploring (Quaranta et al. 2022; Rule et al. 2018b).

Therefore, tools that provide further support to novices, experts, and interdisciplinary programmers to create, document, and visualise notebooks that perform exploratory tasks will be helpful. In this context, our work provides contributions and further insights into the opportunities for software visualisation for computational notebooks.

### Further Tools and Features in Computational Notebooks

Our study has several implications with respect to improving the support for the development and comprehension of computational notebooks. Our qualitative study reveals the needs of data scientists when comprehending and modifying a third-party notebook (refer to Section 6.6.1).

To understand and extend a data science code, a data scientist needs to interact with the existing code and other supplementary information like comments. During the experiment, we found that many participants did not just read but also executed the notebooks by running the cells separately or using the “run-all” option provided by Jupyter. If they encountered an error while executing the notebooks, a majority of the participants looked for cues from comments as well as the data and tried to solve the bug, while a few gave up and proceeded by only reading the notebook. Most errors concerned the variable names, and the participants felt they no longer had the original environment of the variable to fix them. This shows that information from tools that support tracking of history (Kery et al. 2017; Kery and Myers 2018), data flow (Patterson et al. 2017) are likely to be helpful, however, must be presented in a meaningful and compact way in order to avoid overloading the data scientist.

### Supporting (non-linear) Workflow Development in Computational Notebooks

Our linearity analysis has implications for tools that automate the modification of notebooks (refer to Section 5.3). Our method to identify the divergence points can benefit other tools that are targeted towards automated cleaning, splitting, merging, re-ordering, and re-structuring of the existing notebooks for various purposes like readability and replication. Given the prevalence of non-linearity, these tools should also consider the dependencies (e.g., variables used, logical continuity) of the current execution environment of the notebook while modifying its structure. Our method, when integrated within the notebook environment using active learning approaches, can support the developers with automatic identification and documentation of forks with less effort and more accuracy.

### Support in Automation of Analysis

In our experiment, we observed that many participants wanted to add more ad-hoc analyses between the original notebook cells in order to understand the data science task better (e.g., visualising different features of the dataset). Such additions will change the state of the original notebook as presented to them and, if not documented, may lead to loss of information like the author who created the analysis. As a result, tools that support comprehension should also consider and facilitate different modes of collaboration (Nosek 1998). Moreover, the participants in our experiment also exhibited an interest in an assistant that would automatically present them with an initial set of analyses. For example, given a user input and its desired output (Bavishi et al. 2019) synthesised programs for Pandas API. Such support may save effort and time for the user.

### Support for Knowledge Tracking

Due to the vast expansion of data science in the past years, it is difficult for data scientists to keep up with emerging methods and techniques (Kim et al. 2017). We observed this in our experiment as many participants expressed their lack of knowledge about specific methods. This challenge can be overcome by, for example, integrating data science specific named entity recognition tools (Ye et al. 2016) in computational notebook environments. Such a tool can provide support both during development and modification by suggesting alternative methods and techniques that are yet to be explored. The entities can also be linked to other relevant knowledge sources like StackOverflow and GitHub for further support in solving data science tasks.

### Support for Data Science Workflow Documentation

Additionally, our study reveals that participants prefer to have a detailed account of the data science workflow. This account should include, for example, the decisions that influence the selection of certain features and methods among available alternatives and is different from versioning which focuses on low-level code details. The participants’ need also reflects the view from the literature (Gelman and Loken 2013; Steegen et al. 2016) that a record of the explored alternatives that were both successful and unsuccessful (dead-ends) should be documented for verification and future maintenance. Such support for the documentation of the workflow, the rationale of the decisions made, and the dead-ends encountered should be integrated into computational notebook environments to provide a complete account of the narrative.

The software visualisation method developed in this work addresses some of the requirements described above. The workflow information provided in the form of visualisation and annotations and the integration of additional descriptions of the cells along with the orientation cues in our plugin were found to be helpful by the participants in navigating through the code and improving their comprehension.

## Conclusion

In this paper, we presented an empirical analysis of the existence of non-linearity in real-world Jupyter notebooks and proposed a method to visualise workflow information in non-linear data science notebooks to enrich the data science narratives, which has several implications for supporting the development of data science workflows. Our empirical analysis confirms previous preliminary evidence that most real-world notebooks do not follow a linear workflow but instead follow an explorative path that gives rise to forks and dead-ends. A controlled experiment involving our visualisation method showed that presenting data scientists with extra workflow information improves third-party notebook comprehension. The results and insights obtained also call for improvements in computational notebook environments that support efficient programming and presentation of non-linear workflows in computational notebooks for better comprehension. Other obstacles faced during a third-party notebook comprehension reveal insights into the needs of data scientists, and tools addressing the issues found from the user study will likely be received well by the community.

Further studies can be conducted to understand the effects of additional workflow information on a larger set of notebooks that vary in their characteristics (e.g. size and domain). An additional research question that remains unanswered relates to the understanding of the evolution of data science comprehension over time. The design issues that we discovered in the experiment suggest how the visualisation can be improved in terms of usability to better support data science code comprehension and generally improve the user experience while interacting with third-party notebooks and the marg plugin.

## Figures and Tables

**Fig. 15:**
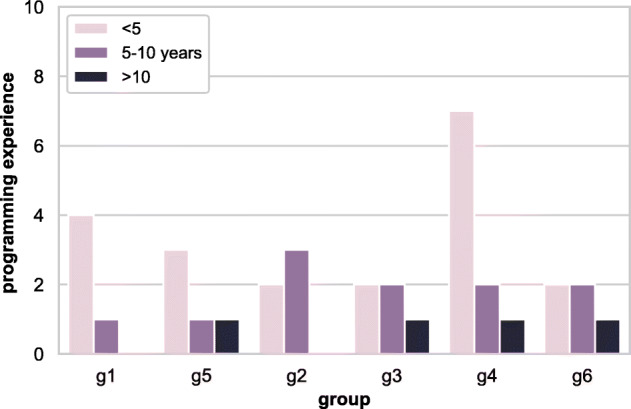
Programming experience among the groups

**Fig. 16:**
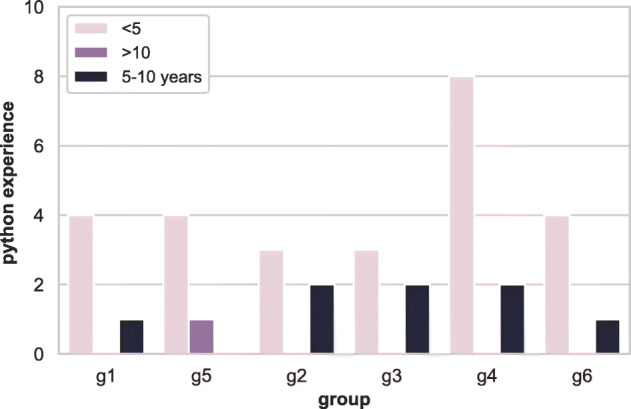
Python experience among the groups

**Fig. 17:**
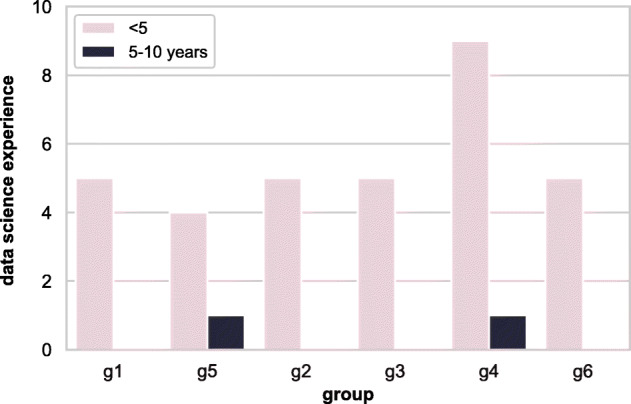
Data science experience among the groups

**Fig. 18:**
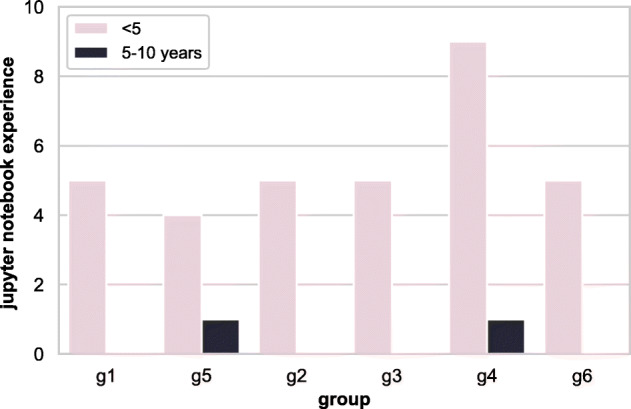
Jupyter notebook experience among the groups

## Data Availability

All data and materials that support the findings of this study are available at the following link: 10.5281/zenodo.7476596.

## Appendix: A

### A.1 Participants Details

Figures 15, 16, 17, and 18 provide more information about the participants’ experience.

### A.2 Comprehension Questions

The list of comprehension questions and its corresponding dimensions are given in Table 5.

**Table 5:** Program comprehension questions and its dimensions

question_id	question	dimension
1	Please share what you think are the 3 main points or takeaways from the solution implemented in the notebook.	−
2	How many features of the dataset are used in the modelling phase that leads to the final solution?	Understanding
3	How many approaches did the author of this code explore while solving the given task?	Understanding
4	What is the modelling technique used for the final solution?	Understanding
5	Why do you think the data scientist created the variable ‘unique_housing_prop’ and for what?	Understanding
6	In which cell did the data scientist create the features that are used in both the ’shifted features - correlation’ method and the ’random forest’ method? (Indicate the answer by copy-pasting the code snippet or by indicating the cell position)	Navigation
7	Can you think of a different modelling technique that could be useful in the analysis?	Modification
8	Please choose a different feature combination (if possible) and re-run all the modelling that has been explored in the notebook. Please include this addition at the bottom of the notebook by adding new cells. Please do not modify/move/delete the existing cells. This is in order to keep the original notebook order. (Do this task in the Jupyter Notebook provided before answering the question below) Indicate the cell position after which you would include your code if you were about to run the notebook in sequential order.	Modification
9	Please indicate the code snippets that are redundant or not useful to arrive at the final solution if any in the original notebook. (Indicate the answer by copy-pasting the code snippet or by indicating the cell position)	Modification

### A.3 Experiment Questions

#### A.3.1 General Questions before the Task

Please answer the next few questions related to your programming and comprehension strategy in general.

1. Gelman et al. (1) found that: “Researcher degrees of freedom can lead to a multiple comparisons problem, even in settings where researchers perform only a single analysis on their data. The problem is there can be a large number of potential comparisons when the details of data analysis are highly contingent on data, without the researcher having to perform any conscious procedure of fishing or examining multiple p-values.”When you implement a data science task, do you try to exhaust all possibilities (or) come up with different alternative solutions that can be forks which may/may not lead to deadends or solutions?*Note:* Fork - A fork in a data analysis task is, given the same data and hypothesis, one way of arriving at a result. Every fork is a decision point in the given task and bases itself on an idea, assumption or a rationale.Deadend - Any fork in a data analysis that does not let us advance to a result (or) ends before we arrive at a result is a dead end. While dead-ends are not necessary to reproduce the final result, they may/may not have led you to the final result by influencing the thought process of the data analyst. It may pave the way for a fork in the data analysis. (1) ’The garden of forking paths: Why multiple comparisons can be a problem, even when there is no “fishing expedition” or“p-hacking” and the research hypothesis was posited ahead of time’ by Gelman et. al
2. What do you think is the methodology (i.e., waterfall, agile, rapid application, Scrum, Lean etc.) of any given data science task?

#### A.3.2 Task Description

Compare housing and rental prices in five major cities on the United States’ west coast. The five major cities in this task are Seattle, Portland, San Francisco, Los Angeles, San Diego.Dataset: https://www.kaggle.com/zillow/zeconRaw data files required for this task are: ‘data/data_dictionary.csv’, ‘data/cities_crosswalk.csv’ and ‘western_states_time_series.csv’ (available in the data folder that we provide you). A description of the variables can be found in ‘data_dictionary.csv’.

The hypothesis to be tested: ”Housing and rental prices in the given five major cities on the United States’ west coast are correlated”. That is, to test: i) whether rental prices are correlated among 5 major cities on the United States’ west coast ii) whether the housing prices are correlated among 5 major cities on the United states’ west coast.

#### A.3.3 Comprehension Questions

Question 1: Please share what you think are the 3 main points or takeaways from the solution implemented in the notebook.Question 2: How many features of the dataset are used in the modelling phase that leads to the final solution?

*Note:* Final solution is the solution that answers the hypothesis question - whether the housing prices among the five cities are correlated and whether the rental prices among the five cities are correlated.Question 3: How many approaches did the author of this code explore while solving the given task? Question 4: What is the modelling technique used for the final solution?

Please choose only one of the following: random forest / shifted features - correlation / correlation - mean squared distance / other

Make a comment on your choice here:Question 5: Why do you think the data scientist created the variable ‘unique_housing_prop’ and for what? Question 6: In which cell did the data scientist create the features that are used in both the ‘shifted features - correlation’ method and the ’random forest’ method? (Indicate the answer by copy-pasting the code snippet or by indicating the cell position)Question 7: Can you think of a different modelling technique that could be useful in the analysis? Question 8: Please choose a different feature combination (if possible) and re-run all the modelling that has been explored in the notebook. Please include this addition at the bottom of the notebook by adding new cells. Please do not modify/move/delete the existing cells. This is in order to keep the original notebook order. (Do this task in the Jupyter Notebook provided before answering the question below)

Note: Indicate the cell position after which you would include your code if you were about to run the notebook in sequential order.Question 9: Please indicate the code snippets that are redundant or not useful to arrive at the final solution if any in the original notebook. (Indicate the answer by copy-pasting the code snippet or by indicating the cell position)

#### A.3.4 General Questions on the Experience

1. Please indicate the comprehension strategy that you followedPlease choose only one of the following: I first read the code completely to have an overview / I skimmed through the code and then, navigated through the different code snippets as needed / Other
2. Did you find it difficult to navigate through the notebook during the task? Please elaborate on the reasons.
3. Did the notebook contain enough information to report accurate answers? Please provide more information about particular cases.
4. Rate your comprehension of the given notebook. Please choose only one of the following: Poor / Fair / Good / Very good / Excellent Make a comment on your choice here:
5. Did you find any other difficulties during this task with respect to data science code comprehension? Please elaborate.
6. Please indicate if you have any other comments.

#### A.3.5 Usability questions on Marg - Narrative Exploration of the Notebook Plugin

1. Please answer the next few questions related to the plugin. First, you are asked about each separate feature in the plugin and then, about the overall plugin.
2. Was the plugin helpful? Please indicate what it was useful for and what it was not/less useful for.
3. Which plugin features you used were the most useful? And which the least useful?
4. What problems did you face in using the plugin?
5. The information about the data science step that each cell is focusing on Please choose the appropriate response for each item: (Strongly disagree / Disagree / Neither agree nor disagree / Agree / Strongly agree)
  - I think that I would like to use this feature frequently.
  - I found the feature unnecessarily complex.
  - I thought the feature was easy to use.
  - I think that I would need the support of a technical person to be able to use this feature.
  - I found the various functions in this feature were well integrated.
  - I thought there was too much inconsistency in this feature.
  - I would imagine that most people would learn to use this feature very quickly.
  - I found the feature very cumbersome to use.
  - I felt very confident using the feature.
  - I needed to learn a lot of things before I could get going with this feature.
6. The information about the forks and deadends Please choose the appropriate response for each item: (Strongly disagree / Disagree / Neither agree nor disagree / Agree / Strongly agree)
  - I think that I would like to use this feature frequently.
  - I found the feature unnecessarily complex.
  - I thought the feature was easy to use.
  - I think that I would need the support of a technical person to be able to use this feature.
  - I found the various functions in this feature were well integrated.
  - I thought there was too much inconsistency in this feature.
  - I would imagine that most people would learn to use this feature very quickly.
  - I found the feature very cumbersome to use.
  - I felt very confident using the feature.
  - I needed to learn a lot of things before I could get going with this feature.
7. Navigation feature in the plugin Please choose the appropriate response for each item: (Strongly disagree / Disagree / Neither agree nor disagree / Agree / Strongly agree)
  - I think that I would like to use this feature frequently.
  - I found the feature unnecessarily complex.
  - I thought the feature was easy to use.
  - I think that I would need the support of a technical person to be able to use this feature.
  - I found the various functions in this feature were well integrated.
  - I thought there was too much inconsistency in this feature.
  - I would imagine that most people would learn to use this feature very quickly.
  - I found the feature very cumbersome to use.
  - I felt very confident using the feature.
  - I needed to learn a lot of things before I could get going with this feature.
8. Overall Marg Plugin Please choose the appropriate response for each item: (Strongly disagree / Disagree / Neither agree nor disagree / Agree / Strongly agree)
  - I think that I would like to use this plugin frequently.
  - I found the plugin unnecessarily complex.
  - I thought the plugin was easy to use.
  - I think that I would need the support of a technical person to be able to use this plugin.
  - I found the various functions in this plugin were well integrated.
  - I thought there was too much inconsistency in this plugin.
  - I would imagine that most people would learn to use this plugin very quickly.
  - I found the plugin very cumbersome to use.
  - I felt very confident using the plugin.
  - I needed to learn a lot of things before I could get going with this plugin.

### A.4 Comprehension Task Answers

The reference set of answers to the comprehension questions (refer to Table 5) is listed by *question_id* in Table 6.

**Table 6:** Answers to the comprehension questions based on the notebook provided to the participants

question_id	answer
1	(question attempted)
2	2
3	3
4	correlation-mean squared distance
5	to be able to determine what kind of property we are dealing with per line
6	cell 25
7	any suitable modelling technique that is not already present in the given notebook
8	(question attempted)
9	All the cells in the fork exploring driver analysis was not used in the final solution.

## Footnotes

1 Narrative and computational narrative are used interchangeably throughout this paper

2 The plugin was implemented as a prototype for experimental purposes providing a static view of the workflow present in the notebook, i.e., we do not consider the updated information if the corresponding notebook is modified and as a result, the visualisation will not be updated.

3 code blocks with order information

4 developed independently from existing visualisations

5 from our previous work on generating an expert annotated dataset of computational notebooks.

6 Agile development “advocates adaptive planning, evolutionary development, early delivery, and continual improvement, and it encourages rapid and flexible response to change” (see https://en.wikipedia.org/wiki/Agile_software_development)

7 proportionately equal to the distribution in DASWOW

8 The experts participated in the task individually and at different times.

9 We also provide the complete list of questions with instructions in the supplementary material.

## Code Availability

All data and materials that support the findings of this study are available in our replication package at the following link: 10.5281/zenodo.7476596.
